# Research on the Relationship Between Cross-Level Motivation, Integrated Emotion, and Virtual Knowledge Community Commitment

**DOI:** 10.3389/fpsyg.2021.563024

**Published:** 2021-07-30

**Authors:** Bing Sun, Hongying Mao, Min Kang

**Affiliations:** School of Economics and Management, Harbin Engineering University, Harbin, China

**Keywords:** motivation level, integrated emotion, community commitment, multiple intermediary, interactive complement

## Abstract

Community commitment is the key to the success of virtual communities. Under the background of virtual knowledge community, based on motivation hierarchy model and integrated emotion theory, this paper takes “motivation-emotion-community commitment” as the main framework, and introduces multiple mediation and regulation functions to establish the relationship model of motivation hierarchy, integrated emotions, and community commitment. The results show that the user motivation follows the hierarchical structure of the layer-by-layer influence from the situational level to the personality level, that is, knowledge-seeking motivation and entertainment-seeking motivation at a situational level will positively affect social- interaction motivation at the contextual level, thereby enhancing user self-efficacy at personality level. Users have abundant integrated emotions toward the virtual knowledge community, namely, satisfaction, attachment, and identity, and such multi-integrated emotional model is more conducive for promoting community commitment of users. At the same time, attachment, identity, and satisfaction have an interactive complement, that is, when satisfaction is low, attachment and identity will complement and strengthen community commitment.

## Introduction

With the development of Internet technology and the rise of the era of big data, the methods of information transmission, knowledge exchange, and knowledge production have undergone tremendous changes. The popularity of intelligent terminals has further promoted knowledge search and knowledge sharing. Virtual knowledge community has become an important platform for people to learn, utilize, produce, share, and acquire knowledge (Kimmerle et al., 2015). The virtual knowledge community is a new type of virtual community with flexibility and creativity, which is guided by knowledge activities and aims at knowledge creation and dissemination (Chen, 2005). With the development of instant messaging technology and the rise of online social services, the form and extension of virtual knowledge communities have undergone important changes, and they are gradually becoming a socialized model with more types of shared knowledge, more participants, and more stable relationships (Liu and Deng, 2013). This model makes the community present the characteristics of diversified topics, diversified knowledge types, original knowledge, and user sociality. At present, typical virtual knowledge communities mainly include Douban, Zhihu, and Baidu Tieba. Their existence meets individualized needs for knowledge and social needs for interpersonal relationships of people. Due to the anonymity of virtual knowledge community users and the obstacles of time and space, users can choose to join or stay in a community or leave at any time according to their own situation (Wang et al., 2019). In fact, although there are many successful communities with high user participation, there are quite a few communities with low user participation, too many “dive” users, and serious user loss. Therefore, in order for the community to survive and function effectively for a long time, it is necessary not only to attract new users, but also to maintain old users to stimulate and ensure their continued participation behavior (Muñiz and O'Guinn, 2001). Previous studies have shown that continuous participation behavior of the user is an interactive process, in which the core role is community commitment of the user (Brodie et al., 2013). It can be seen that community commitment is the key factor to determine the success of a virtual knowledge community.

The concept of commitment originates from the study of organizational behavior. Becker (1960) first defined organization commitment, believing that organization commitment refers to the behavior intention of maintaining a continuous relationship between individuals and organizations in an organization, explaining the phenomenon that individuals are willing to stay in the organization with the increase of their unilateral input to the organization. Community is an organization. Community commitment derives from organizational commitment, which refers to the intention of maintaining a long-term relationship between users and communities in the community (Zhou et al., 2012). At present, the academia mainly studies virtual community commitment of the user from two aspects of psychological driving factors and emotional cognition, that is, paying attention to and explore how continuous participation behavior of users is driven by motivation mechanism and which emotions are affected. Regarding the driving mechanism of community commitment, some scholars started to study the motivation factors of different types of virtual communities and pointed out the intrinsic motivation of online communities (Hau and Kim, 2011), instrumental motivation, functional motivation, and social motivation of virtual communities (Dholakia et al., 2004), the internal and external motivations of transactional virtual communities (Sun et al., 2012), as well as the motivations of entertainment seeking, information seeking, and community interaction of live media communities (Hilvertbruce et al., 2018) have a positive impact on community commitment. Other scholars studied the differences in the influences of different motivations on community commitment, pointing out that extrinsic motivation and intrinsic motivation have different influences on the continuous participation of users (Zhang et al., 2017). Through meta-analysis, Wu and Lu (2013) found that in an entertainment information system, intrinsic motivation plays a stronger role than extrinsic motivation. In the practical information system, extrinsic motivation plays a stronger role. In terms of research on the impact of affective cognition on virtual community commitment, many scholars pointed out that satisfaction is an important emotion that affects the community commitment of users (Tang and Chiang, 2010). Meanwhile, some scholars have also confirmed that attachment is an important emotion that affects the commitment of users to virtual communities (Rever, 1972; Limayem and Hirt, 2003; Carroll and Ahuvia, 2006).

From the above literature, although the research on the motive-driven mechanism and the emotional cognition influencing factors of virtual community commitment of users is increasingly widespread, there are still the following shortcomings: First, scholars at home and abroad mostly focus on virtual community, but seldom pay attention to virtual knowledge community. Second, scholars mostly study motivation as a single concept or put different types of motivation in the same dimension, and seldom analyze community commitment from the perspective of motivation level. Third, most scholars regard satisfaction in emotional cognition as a direct influencing factor of community commitment, neglecting the complementary role of multiple and integrated emotions of users (such as satisfaction, attachment, and identity) on community commitment. Finally, scholars only pay attention to the individual impact of motivation or emotion on community commitment, and pay less attention to the joint impact of motivation and emotion on community commitment, which may exist.

So, what is the relationship between different motivations, integrated emotions, and community commitments in a virtual knowledge community? Is it possible to explain the impact of different types of motivations of users on virtual knowledge community commitments through a cross-level perspective? In the process, what is the role of multiple integration emotions? Does integration emotion and different motivations have a joint effect? What is their mechanism of action for community commitment? To solve the above problems, this paper is based on the motivational level model and the integrated sentiment theory, with “motivation-emotion-community commitment” as the main framework, and conducts in-depth research on the mechanism of community commitment by different levels of motivation and multiple integration emotions. In order to reveal the complex relationship between motivation, multi-integration emotion, and community commitment, it provides theoretical reference and decision-making for the sustainable development and scientific management of the virtual knowledge community.

## Theoretical Basis and Research Hypothesis

### Motivation Hierarchy Model

The hierarchy of motivation has always been the dominant structure in the psychology literature (Austin and Vancouver, 1996). Sheldon et al. (2011) pointed out that the integration of multiple levels of motivation analysis can properly describe the importance of human achievement of goals. To this end, Vallerand (1997) proposed the Model of Intrinsic and Extrinsic Motivation (HMIEM) based on self-determination theory, which provides a hierarchical perspective on human motivation and enables researchers to analyze and understand the determinants and outcomes of motivation types at different levels. This model considers three levels of motivation, namely, situational level, contextual level, and personality level. Among them, the motivation at the situational level refers to the motivation that the individual has for engaging in the current activities, and it is crucial to better understand the initial behavior of the individual; the motivation at the contextual level refers to the general motivation orientation of the individual toward a specific social environment, which mainly includes work, leisure, and interpersonal relationships; the motivation at personality level refers to the motivation of autonomously oriented behavior determined by individual personality, which will have a relatively lasting impact on individual behavior (Vallerand, 1997). The model reveals the interaction between motivations at different levels, that is, motivation at one level is influenced by motivation at another level. Specifically, motivation at the situational level will influence motivation at the contextual level and personality level in turn, or will influence motivation from personality level to situational level in reverse step by step (Vallerand and Lalande, 2011).

However, when motivation is brought into the environment of the virtual knowledge community, the hierarchical structure is ignored. Most studies investigated the motivation of users to participate in virtual communities, but the results were limited to motivation types without considering the possibility of hierarchy (Xu and Li, 2015; Feng and Ye, 2016; Guan et al., 2018). In order to better study the impact of different motivations on the commitment of users to the virtual knowledge community, drawing on the research results of Vallerand (1997), this paper argues that motivation of users to participate in virtual knowledge community mainly includes: knowledge-seeking motivation and entertainment-seeking motivation at the situational level, social-communication motivation at the contextual level, and user self-efficacy at the personality level. Dholakia et al. (2004) believed that the original motivation starts with self-interest. In the virtual knowledge community, the motivation of self-interest is embodied in the motivation of knowledge seeking and entertainment seeking at the situational level (Jin et al., 2010). If these initial motivations of users are gradually strengthened, the motivations of the adjacent levels will also be gradually strengthened. Therefore, the layer-by-layer influence relationship from situational level to personality level is more suitable for understanding the motivation of commitment of users to virtual knowledge community (David and Shapiro, 2008).

### Integrated Emotion

Gray et al. (2002) pointed out that emotions have a strong effect on behavioral intentions. The continuous participation of users should follow the logic of long-term emotional effects (Raies et al., 2015). It can be seen that emotions have an important impact on the continuous participation behavior of users (Limayem and Hirt, 2003). After Cohen et al. (2008) first proposed the concept of integrated emotion, Cohen et al. defined integrated emotion: integrated emotion refers to emotional induction that has a direct relationship with a judgment or decision object and a real experience, this emotional induction is a true, perceived, or imagined integrated attitude toward the object. Considering that users participation in the virtual knowledge community cannot do without the real experience emotions of the community, this article introduces integrated emotions to study its impact on community commitment.

Previous studies have pointed out that users have multiple emotions, such as satisfaction, attachment, and identity, and positive emotions will better promote the sustainable participation behavior and community commitment of virtual community users (Raies et al., 2015; Tseng et al., 2017). Satisfaction, attachment, and identity all belong to the category of integrated emotions (Pan and Huang, 2017). All three are emotions generated based on interactive stimuli with the virtual community. First, these three emotions are independent of each other and have different emotional patterns. Satisfaction is a direct reactive emotion that the perceived value reaches or exceeds the expected value of the user; attachment is a stable endogenous relationship dependence formed during the interaction between the user and the virtual community; similarly, identity is a deeper relationship between the user and the virtual community, and it should be based on satisfaction. Finally, attachment and identity of the user are useful complements to satisfaction. The attachment and identity are the extension and strength of the emotional relationship of the user, which reflects the psychological and social needs of the user beyond the functional needs of the virtual community (that is, satisfaction), and is a more durable and stable performance of the relationship of the user with the virtual community. Therefore, attachment and identity are complementary complements of satisfaction. As Jin et al. (2010) pointed out, satisfaction and psychological attachment emotions (such as attachment and identity) jointly determine the intention of the user to continue using.

### Research Hypothesis

#### Interaction Between Different Levels of Motivation

As mentioned earlier, situational motivations include knowledge-seeking motivation and social-interaction motivation. Among them, knowledge-seeking motivation refers to the motivation that users desire to obtain knowledge information from the virtual knowledge community (Dholakia et al., 2004); social-interaction motivation refers to encouraging users to establish and maintain contact with other users in order to obtain social benefits from social support, friendship, and intimacy motivation (Dholakia et al., 2004). People are born with a behavioral tendency, namely information search and seeking knowledge, which shows that people have a strong demand for knowledge (Maslow, 1943; Vandenbosch and Huff, 1997). Most scholars have confirmed that the virtual knowledge community has become an important way to seek knowledge (Maslow, 1943; Feng and Ye, 2016; Guan et al., 2018). Knowledge-seeking motivation is not only the most mentioned motivation for users to participate in the virtual community (Tseng et al., 2017), but also the initial motivation for users to participate in the virtual community (Maslow, 1943). Although the motivation for seeking knowledge is limited in emotional stimulation, it can stimulate social motivation, especially social-communication motivation (Chiu et al., 2006). Specifically, when users search for information or find answers in the virtual knowledge community based on knowledge-seeking motivation, sometimes they can directly find information or answers, and sometimes they are difficult to find. At this time, users with social-interaction motives tend to post questions in the community in order to reply. When these questions are answered, social interaction also occurs. Correspondingly, knowledge-seeking motivation stimulates knowledge- seeking behavior, and knowledge-seeking behavior is an effective way to trigger social-communication motivation and develop social relationships (Ma and Chan, 2014). This shows that knowledge-seeking motivation can enhance the social -interaction motivation of members of the virtual knowledge community. Therefore, this article proposes the following hypothesis:

H1: Knowledge-seeking motivation of users in the virtual knowledge community has a positive impact on their social-interaction motivation

Entertainment-seeking motivation is the motivation to inspire users to get fun and relax by browsing information in the virtual community (Dholakia et al., 2004). Entertainment is an important incentive for using virtual communities (Dholakia et al., 2004). The main motivation for entertainment-seeking motivation is the entertainment value provided by the virtual knowledge community, which is usually reflected in leisure activities, such as playing games or having pleasant conversations. Unlike knowledge-seeking motivation with knowledge-oriented goals, entertainment-seeking motivation is not to achieve a specific goal, but to pass the time or meet leisure needs. Therefore, situational level entertainment seeks opportunities to encourage users to participate in discussions, sweepstakes, contests, and games in the virtual community. This not only meets the entertainment needs of the user, but also leads to higher levels of discussion and games with other users' social-interaction needs (Madupu and Cooley, 2010). That is to say, although a user joins the virtual knowledge community initially to satisfy entertainment needs, entertainment activities shared with other users with similar interests will generate and strengthen motivation for social interaction. Tseng also pointed out that in the virtual brand community, the entertainment-seeking motivation of users can stimulate social-communication motivation (Tseng et al., 2017). Therefore, the following hypothesis is proposed:

H2: Motivation of users for seeking entertainment in a virtual knowledge community has a positive impact on their social-interaction motivation

Self-efficacy is a way for individuals to achieve internal satisfaction. It refers to enhancing the confidence and self-esteem of users by sharing valuable information with others in a virtual community (Constant et al., 1996). Self-efficacy is one of the core concepts of social cognition theory and belongs to the intrinsic motivation of the personality level. The social-communication motivation is a way to show social support (Barrera and Ainlay, 1983), which strengthens the social relationship among members of the virtual knowledge community. In a virtual knowledge community, social interaction driven by social-communication motivation can help users increase their influence. Research on the operation and management of virtual communities shows that social-communication motivation is an important factor in determining the success of the community, and it can increase the confidence of community users to share valuable knowledge (Sindhav et al., 1998). Research shows that, on the one hand, through community interaction based on social-interaction motivation, users can obtain information and experience about a certain problem, thereby realizing a reassessment of self-efficacy (Bandura, 1986). On the other hand, participating in the discussion of the same knowledge issue and sharing valuable information with other users will also increase the confidence and self-esteem of the user. In particular, positive comments made by other users in the community can enhance the sense of social identity of the user (Dholakia et al., 2004; Yu et al., 2010). It can be seen that user interaction based on social-communication motivation will bring about the recognition of the individual value and the promotion of social status (Fuller, 2006). Therefore, social-communication motivation provides an effective way to improve self-efficacy. Therefore, the following hypothesis is made:

H3: The social-interaction motivation of users in the virtual knowledge community has a positive impact on their self-efficacy

#### The Impact of Personality Level Self-Efficacy on Satisfaction

Bandura and Cervone (1983) studied the sense of self-efficacy from the perspective of result expectation and performance expectation, and believed that when an individual believes that he has the ability to complete a certain behavior, and through this behavior can achieve the expected result, the user will receive corresponding benefits or realize self-worth, thereby enhancing user satisfaction. As Maslow's hierarchy of needs theory says, people all have self-fulfilling value needs, so users of virtual knowledge communities tend to share valuable information with other users or play their role in the community to prove their ability and achieve self-worth. When the needs of users of the virtual knowledge community to realize self-worth are satisfied, their satisfaction will increase. Boudreau also pointed out that users with higher self-efficacy in the community website are not only more likely to be respected by other users, but also promote own satisfaction of users (Boudreau et al., 2010). In addition, users with a high sense of self-efficacy will think that the value of the community is higher (Hopp et al., 2018), so these users are willing to meet their information needs or emotional needs through frequent interaction with other users in the community, thereby improving their satisfaction. Accordingly, this study proposes the following hypothesis:

H4: The self-efficacy of users in the virtual knowledge community has a positive impact on their satisfaction

#### Mutual Influence Between Integrated Emotions and Interactive Supplementary Influence on Virtual Knowledge Community Commitment

Drawing on the psychological perspective, users will have emotional attachments to the virtual community because their needs are met (Raies et al., 2015). Bhattacherjee (2001) also found through research that satisfaction can enhance the attachment of users to virtual communities. It can be seen that satisfaction will promote the attachment of users to the virtual community. In the virtual knowledge community, network services provide users with relevant knowledge information to solve problems to increase the social satisfaction of the user, or set the content of the entertainment experience of the user to satisfy the sensory and psychological pleasure of the user. According to the theory of attachment determination, these social network services improve the use of virtual knowledge communities of users and meet their ability needs, thereby promoting users to form a stable endogenous relationship of dependence, that is, attachment. At the same time, according to the theory of social identity, the virtual knowledge community provides users with a platform for mutual communication. Users can gather together according to their own value needs or common interests and hobbies. The improvement of community identity will bring users a better psychological experience and value, make users perceive more fun and pleasure, these psychological and emotional benefits will greatly enhance user satisfaction, make the community more attractive, and thus help users form a consistent sense of social belonging to the virtual community, that is, to form a sense of identity. It can be seen that satisfaction will enhance the identity of users in the virtual knowledge community. Therefore, this article proposes the following hypotheses:

H5a: User satisfaction in the virtual knowledge community has a positive effect on their attachment
H5b: User satisfaction in the virtual knowledge community has a positive impact on their identity

The emotional paradigm has a strong influence on virtual community commitment (Raies et al., 2015). The premise of community commitment is psychological and emotional connection. The main method of constructing the psychological and emotional connection of community commitment is to use the difference between revenue and cost to measure whether the expected value is exceeded to determine whether the user has made a commitment. Therefore, community commitment can take effect through satisfaction (Tseng et al., 2017). Many scholars have conducted research on the impact of satisfaction on the willingness of users to participate and behavior. They pointed out that satisfaction is the main reason for the willingness of the virtual community users to contribute knowledge (Ma and Chan, 2014), the continuous use of information systems (Bhattacherjee, 2001), and the virtual brand community commitment (Tseng et al., 2017). In the virtual knowledge community, users mainly have functional needs or social needs. When a certain demand is met, the user receives benefits from the virtual community. When the perceived value reaches or exceeds the expected value, the user will feel satisfied, and then have the intention to maintain long-term contact with the virtual knowledge community, that is, community commitment. It can be seen that user satisfaction is a positive influencing factor of community commitment. Therefore, the following hypothesis is proposed:

H6: User satisfaction in the virtual knowledge community has a positive impact on community commitment

With the continuous emergence of virtual communities in various fields, frequent visits to virtual communities can make users have a sense of attachment to the community and affect community commitment through the sense of attachment (Limayem and Hirt, 2003). Attachment is an important link for users to maintain a lasting connection with the community (Carroll and Ahuvia, 2006), which is expressed as: attachment is highly correlated with user behavior, making users have a strong tendency (Rever, 1972), users with strong attachment are more willing to pour time, energy, and other resources to meet in the virtual knowledge community their own functional and social needs, and then increase the frequency of use of virtual knowledge communities. In other words, users with stronger attachment feelings have a higher willingness to maintain long-term relationships with the virtual community (Drigotas and Rusbult, 1992). Further, when negative information or destructive behaviors in the virtual community lead to relatively low user satisfaction, attachment feeling is an important basis for users to make decisions (Pan and Huang, 2017). That is, a sense of attachment will make it easier for users to forgive and understand community errors (Mccullough et al., 1998), and ignore some negative information in the community (Ahluwalia et al., 2000), and then choose to continue to stay in the virtual community, even when other users make destructive actions against the community. At times, people with a strong sense of attachment can curb their impulsive reactions, thereby avoiding some destructive behavior (Drigotas and Rusbult, 1992). It can be seen that the reason why users are still willing to maintain a long-term relationship with the virtual knowledge community in the case of low satisfaction is that the sense of attachment has a strong supplementary effect on community commitment. Therefore, the following hypotheses are proposed:

H7: The sense of attachment of users in the virtual knowledge community has a positive impact on community commitment
H7a: In the case of low satisfaction, attachment feeling supplements and reinforces the commitment of the virtual knowledge community

In a virtual community, whether a satisfied user stays in the community depends on the behavior of other users in the community (Hsu et al., 2004). If the user regards other users as opportunists or disagrees with the views and opinions of other users, he will be reluctant to continue using the community (Yen and Yan, 2013). Empirical studies have shown that identity promotes user participation behavior (Dholakia et al., 2004; Bagozzi and Dholakia, 2006b). The stronger the identity of the user, the stronger his feelings about the emotional relationship in the community, and the more he will strengthen the dependence and emotional input, the more willing to maintain long-term interaction with the community. Based on a strong sense of identity, six users are willing to join in emotional communication with other users, and are more likely to maintain community commitment through continuous interaction (Usoro et al., 2007). Algesheimer et al. (2005) also pointed out that the higher the sense of identity of the community user, the lower the pressure of community norms perceived by users and the more willing users are to participate in community activities. Conversely, if there is no strong sense of identity, they can easily switch to another alternative community by clicking (Kim et al., 2008). Further, in the case of low satisfaction, identity is also an important basis for user decision-making (Pan and Huang, 2017). In other words, user identity has a positive effect on resisting negative information and complaints of other users, so that when user satisfaction is relatively low, users are still willing to form a close connection and continuous participation relationship with the community (Pan and Huang, 2017). In addition, users with high sense of identity generally belong to the category of loyal users, and they tend to pay more attention to the long-term development of the participating communities. Even when they are dissatisfied with the community, users often ignore their own interests and provide the community with a way to improve the current operation good advice. It can be seen that in the virtual knowledge community, the sense of identity supplements and strengthens the community commitment. Therefore, the following hypotheses are proposed:

H8: Identity of the users in the virtual knowledge community has a positive impact on community commitment
H8a: In the case of low satisfaction, the sense of identity supplements and reinforces the commitment of the virtual knowledge community

**Figure 1:**
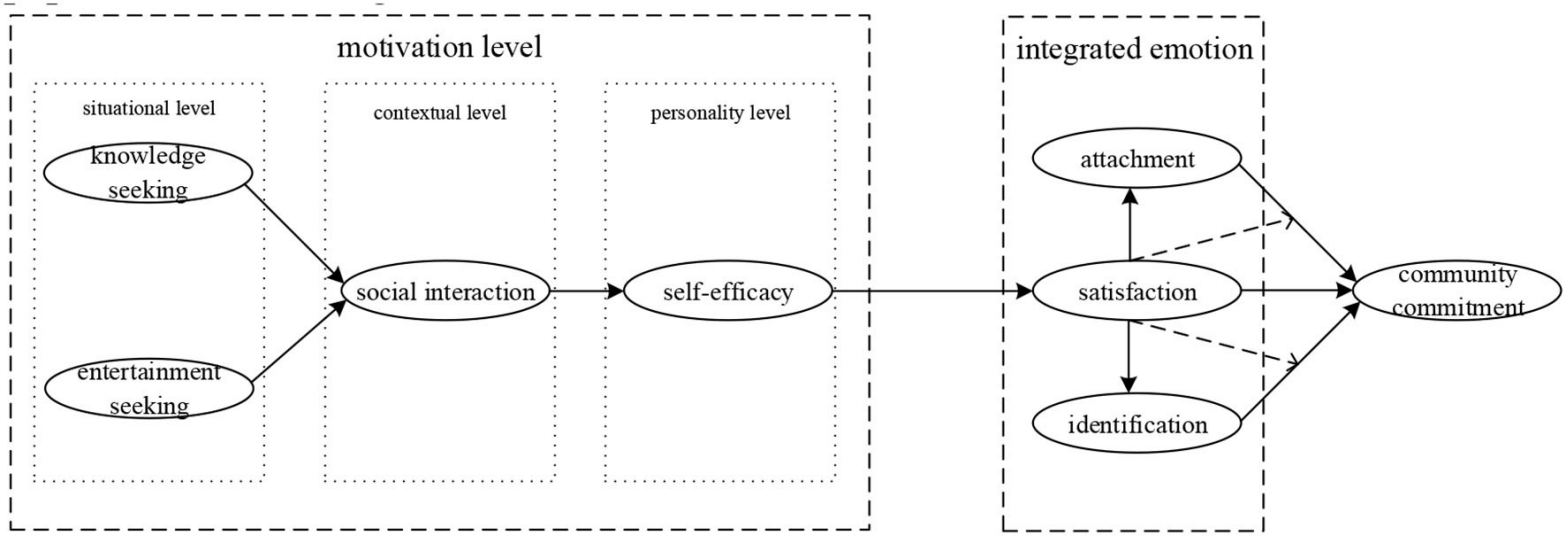
The theoretical model.

Based on the above theoretical assumptions, the theoretical model constructed in this paper is shown in Figure 1.

## Research Design

### Variable Measurement

This article involves eight concepts of knowledge-seeking motivation, entertainment-seeking motivation, social-interaction motivation, self-efficacy, satisfaction, attachment, identity, and community commitment. The basic principle of the scale design is: on the basis of referring to the relevant literature, according to the characteristics and scenarios of the virtual knowledge community to make adaptive modifications.

For the measurement of knowledge-seeking motivation, entertainment-seeking motivation, and social-interaction motivation, this paper mainly refers to the scale of Dholakia et al. (2004). Among them, under the knowledge-seeking motivation variable, there are four measurement items such as acquiring information, learning to do things, solving problems, and generating new ideas; under the variables of entertainment-seeking motivation, four measurement items are set: pursuit of entertainment, curiosity, relaxation, and passing time; three measurement items are set up under the social-communication motivation variable, such as establishing contact, maintaining contact, and impressing. For the measurement of the self-efficacy of user, this article mainly refers to the scale of Hsu et al. (2007). Under this variable, four measurement items are set, including confident knowledge provision, skilled knowledge provision, ability to respond to messages and comments, and confident knowledge sharing.

For the measurement of satisfaction, this article draws on the Davis scale to set up four measurement items such as income gain, functional suitability, interest conformity, and overall satisfaction (Davis et al., 1989); the measurement of attachment feeling mainly refers to the Thomson et al. (2005) scale, which sets up five community feeling measurement items such as mood improvement, closeness, cherishment, attachment, and intimacy; for the measurement of identity, refer the scale of Haumann et al. (2014), which sets up three measurement items, such as the objection to the poor community evaluation, the degree of attention to the community evaluation, and the sense of belonging.

For the measurement of community commitment, we mainly refer to the scales of Shukla et al. (2016) and Mathwick et al. (2008), and set six measurement items, including the importance of the community to users, the care for the development of the community, the difficulty to the community, the sense of life confusion when leaving the community, the sense of uneasiness when leaving the community, and the willingness to maintain an indefinite relationship with the community.

All the above measurement items are measured by the five-level scale of Likert, of which five indicates complete approval and one indicates complete disagreement. Table 1 lists the constructs and measures.

**Table 1:** Constructs and measures.

**Construct**	**Item**	**Measures**	**References**
Knowledge-seeking motivation	KS1	I visit the virtual knowledge communities for information	Dholakia et al., 2004
	KS2	I visit the virtual knowledge community to learn how to do things	
	KS3	I visit the virtual knowledge community to solve the problems encountered	
	KS4	I visit the virtual knowledge community to generate new ideas	
Entertainment-seeking motivation	ES1	I visit the virtual knowledge community for entertainment	Dholakia et al., 2004
	ES2	I visit the virtual knowledge community to play	
	ES3	I visit the virtual knowledge community to relax	
	ES4	I visit the virtual knowledge community to pass the time when I am bored	
Social-interaction motivation	SI1	I visit the virtual knowledge community to work with others	Dholakia et al., 2004
	SI2	I visit the virtual knowledge community to connect with others	
	SI3	I visit the virtual knowledge community to keep in touch with others	
Self-efficacy	SE1	I am confident to provide knowledge in the virtual knowledge community	Hsu et al., 2007
	SE2	I am confident to share knowledge in written form in the virtual knowledge community	
	SE3	I have the ability to respond to messages and comments of other people in the virtual knowledge community	
	SE4	I am confident to discuss and share knowledge with others in the virtual knowledge community	
Satisfaction	S1	This virtual knowledge community can bring me benefits	Davis et al., 1989
	S2	I am very satisfied with the functional services provided by the virtual knowledge community	
	S3	The content of this virtual knowledge community fully meets my interests	
	S4	On the whole, I am very satisfied with the virtual knowledge community	
Attachment	A1	When using this virtual knowledge community, my mood will be very good	Thomson et al., 2005
	A2	I cherish this virtual knowledge community	
	A3	This virtual knowledge community has a close relationship with me	
	A4	I have a sense of attachment to this virtual knowledge community	
	A5	When people belittle this virtual knowledge community, I feel that I am belittle myself	
Identity	I1	When others say that this virtual knowledge community is not good, I feel unhappy	Haumann et al., 2014
	I2	When I discuss this virtual knowledge community, I usually use “we” instead of “them”	
	I3	I want to know what other people think of this virtual knowledge community	
Community commitment	CC1	This virtual knowledge community has great personal meaning to me	Shukla et al., 2016; Mathwick et al., 2008
	CC2	I am very concerned about the fate of this virtual knowledge community	
	CC3	It is hard for me to leave this virtual knowledge community now, even if I want to.	
	CC4	If I decide to leave this virtual knowledge community, my life will be disrupted	
	CC5	If I leave this virtual knowledge community now, I will feel guilty and uneasy	
	CC6	I intend to maintain an indefinite relationship with this virtual knowledge community	

### Data Collection

For the purpose of research, the main target of this article is the users of the virtual knowledge community. The study was approved by the respondents. Before the start of the study, we fully explained the purpose and method of the study to the participants, so as to ensure the security and protection of personal information, and ensure that the participants can withdraw from the study at any time without damage. After obtaining the informed consent of the participants, all the data were properly obtained. In order to enhance the accuracy and reliability of the research results, this article obtains the required questionnaire data through online surveys. From July to October 2019, a total of 336 questionnaires were collected through WeChat group links, Tencent QQ group links, Weibo posting links, major well-known knowledge community forums, and questionnaire star websites. Based on the filtering problem, this article eliminated some invalid samples, and finally got a total of 293 valid questionnaires. The questionnaire validity rate was 87.2%. Table 2 presents the sample demographics characteristics. It can be seen from Table 2 that in the sample, male users accounted for 53.92%, and the majority were users aged 31–40 (42.32%), followed by users aged 21–30 (40.27%), and users with a university degree (66.89%) were mainly, it is consistent with the netizen attribute structure in the 43rd survey results released by China Internet Network Information Center (CNNIC) in 2019, while ensuring the representativeness of the questionnaire survey. Judging from the characteristics of the research samples, the knowledge-based young and middle aged are the main user groups of the virtual knowledge community. In addition, we found that most users will use multiple virtual knowledge communities at the same time, and the vast majority of users tend to use Douban (53.24%) and Zhihu (74.06%), which is also consistent with the characteristics that Douban and Zhihu are high-quality Question and Answer communities of Chinese Internet. Among the 293 users, users who have used the virtual knowledge community continuously for <1 year accounted for 13.65%, users for 1–2 years accounted for 36.86%, users for 3–4 years accounted for 31.74%, and users for more than 4 years accounted for 17.75%, it can be seen that, with the exception of users who have used the virtual knowledge community within 1 year, the number of users who continue to use the virtual knowledge community has increased over time and has shown a decreasing trend. Therefore, it is imperative to study the influencing factors of community commitment of users.

**Table 2:** Demographic characteristics.

**Variables**	**Category**	**Frequency**	**Percentage**	**Variables**	**Category**	**Frequency**	**Percentage**
Gender	Male	158	53.92	Which virtual knowledge communities to use (multiple choice)	Douban	156	53.24
	Female	135	46.08		Zhihu	217	74.06
Age	<20 years	14	4.78		Study class related post bar	145	49.49
	21–30 years	118	40.27		Muchong Forum	100	34.13
	31–40 years	124	42.32		National People's Congress Economic Forum	51	17.41
	>41 years	37	12.63		Other virtual knowledge communities	59	20.13
Education	High school or below	23	7.85	Time for continuous use of virtual knowledge community	<1 year	40	13.65
	College	40	13.65		1–2 years	108	36.86
	University	196	66.89		3–4 years	93	31.74
	Graduate school or above	34	11.6		>4 years	52	17.75

## Results and Discussion

The model built in this research is relatively complex, there are multiple independent variables and intermediary variables, so it is suitable to use partial least squares for model testing. At the same time, considering that compared to structural equation modeling software such as AMOS and LISREL, Smart PLS software is more suitable for the analysis of relatively complex models with small and medium-scale samples, so this study uses SmartPLS 2.0 software as a statistical analysis tool to verify the effectiveness of the model and test the hypothesis. In addition, in order to rule out the influence of common method variation, this paper uses single factor test of Harman to check the common method deviation. The test results show that the largest variable contribution rate of a single factor without rotation is 30.880%, <40%, indicating that there is no homology deviation.

### Reliability and Validity Test

Reliability tests include α coefficient test of Cronbach and combined reliability (CR) test. It can be seen from Table 3 that the *α* coefficients of Cronbach of each construct in this article are all >0.7, and the CR is <0.8, indicating that the scale has good reliability.

**Table 3:** Reliability and convergence validity.

**Variable**	**Items**	**Cronbach'sα**	**CR**	**AVE**
Knowledge seeking	4	0.825	0.884	0.656
Entertainment seeking	4	0.652	0.882	0.652
Social interaction	3	0.765	0.865	0.681
Self-efficacy	4	0.845	0.896	0.683
Satisfaction	4	0.812	0.877	0.640
Attachment	5	0.821	0.875	0.583
Identification	3	0.704	0.835	0.628
Community commitment	6	0.865	0.898	0.596

In this paper, the validity test is conducted through convergence validity and differentiation validity. From Table 3, we can see that the average variance (AVE) of each latent variable is >0.5, indicating that each variable has a higher convergence validity. At the same time, as can be seen from Table 4, the square root of the AVE value of all latent variables (bold numbers in the table) is greater than the correlation coefficient between latent variables off the diagonal (non-bold numbers in the table), indicating that the measurement model has a good distinguishing validity.

**Table 4:** Distinguish between validity and latent variable correlation matrix.

	**Knowledge seeking**	**Entertainment seeking**	**Social interaction**	**Self-efficacy**	**Satisfaction**	**Attachment**	**Identification**	**Community commitment**
Knowledge seeking	**0.810**							
Entertainment seeking	0.213	**0.807**						
Social interaction	0.617	0.396	**0.825**					
Self-efficacy	0.224	0.242	0.470	**0.826**				
Satisfaction	0.170	0.175	0.275	0.462	**0.800**			
Attachment	0.439	0.306	0.507	0.293	0.443	**0.764**		
Identification	0.355	0.260	0.491	0.407	0.485	0.633	**0.792**	
Community commitment	0.286	0.270	0.539	0.471	0.516	0.608	0.606	**0.772**

### Structural Model Test

**Figure 2:**
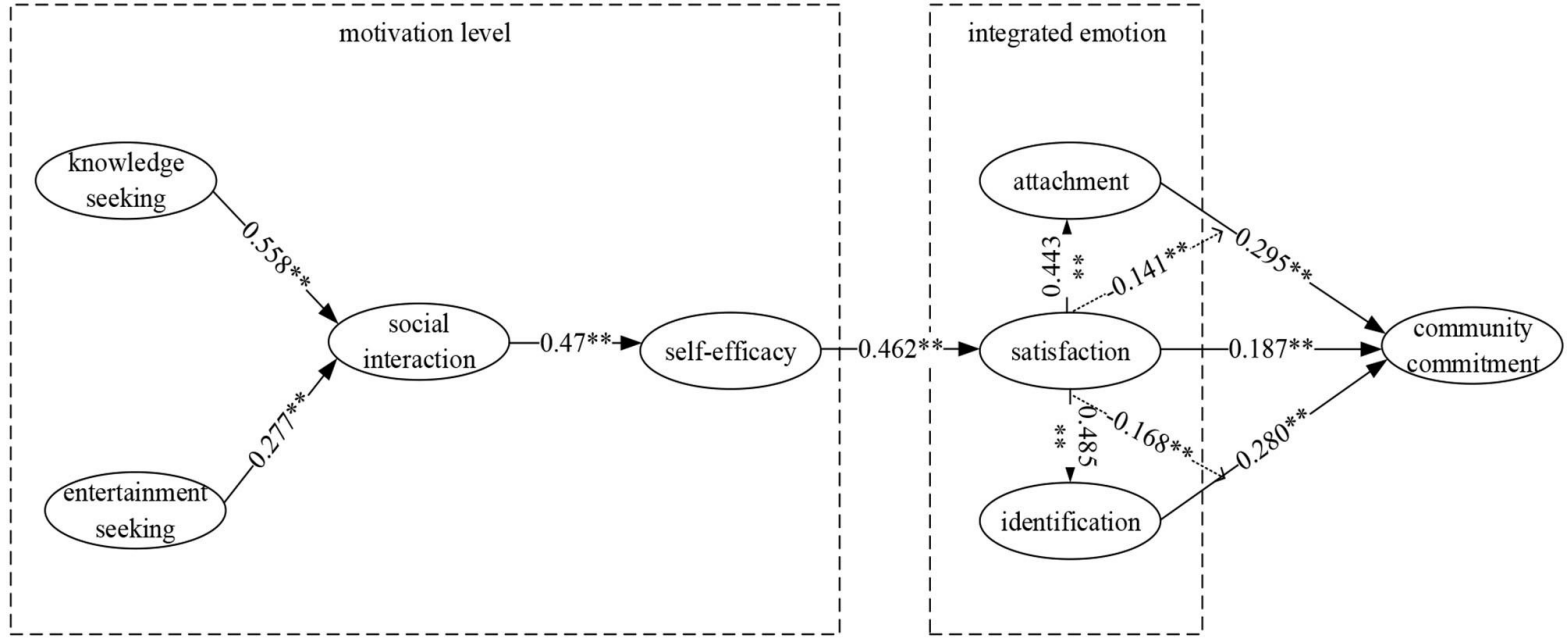
Model test path coefficient. ^**^p < 0.01.

In this study, SmartPLS2.0 software was used to calculate the path coefficients, and the Bootstrapping method was used to iterate 5,000 times to calculate the significance of the coefficients. The path coefficient test results of the model are shown in Figure 2 and Table 5. The basis of theoretical model evaluation is to determine the value of coefficient *R*^2^. The community commitment *R*^2^ in this study is 0.565, which satisfies the requirement that the *R*^2^ in the field of behavior is higher than 0.2 (Hair et al., 2011), indicating that the theoretical model constructed in this paper has a reliable fitting effect. In addition, the goodness of fit (Gof) of the model is 0.439, which is higher than the recommended standard of 0.36.

**Table 5:** Path coefficient *T*-test results.

	**Original sample (O)**	**Sample mean (M)**	**Standard deviation (STDEV)**	**Standard error (STERR)**	**T statistics (|O/STERR|)**
Knowledge seeking -> social interaction	0.558	0.558	0.038	0.038	14.836
Entertainment seeking -> social interaction	0.277	0.279	0.047	0.047	5.841
Social interaction -> self-efficacy	0.470	0.472	0.046	0.046	10.170
Self-efficacy -> satisfaction	0.462	0.464	0.042	0.042	10.997
Satisfaction -> community commitment	0.187	0.186	0.051	0.051	3.653
Satisfaction -> attachment	0.443	0.447	0.051	0.051	8.664
Attachment -> community commitment	0.295	0.297	0.057	0.057	5.207
Attachment ^*^ satisfaction -> community commitment	−0.141	−0.136	0.071	0.071	1.990
Satisfaction -> identification	0.485	0.488	0.049	0.049	9.990
Identification -> community commitment	0.280	0.280	0.062	0.062	4.523
Identification ^*^ satisfaction -> community commitment	−0.168	−0.177	0.065	0.065	2.580

First, the test results show that knowledge-seeking motivation (*β* = 0.558, *p* < 0.01) and entertainment-seeking motivation (*β* = 0.277, *p* < 0.01) have significant positive effects on social-communication motivation. Therefore, hypothesis 1 and 2 are supported. Second, hypothesis 3 proposed that social-interaction motivation has a positive effect on self-efficacy. The results confirmed this (*β* = 0.470, *p* < 0.01). Again, the empirical results show that self-efficacy also has a significant positive effect on satisfaction (*β* = 0.462, *p* < 0.01), and hypothesis 4 is supported. At the same time, the empirical results show that the path coefficients of satisfaction and attachment (*β* = 0.443, *p* < 0.01) and identity (*β* = 0.485, *p* < 0.01) are significant. Therefore, hypothesis 5a and 5b are both supported. Finally, satisfaction (*β* = 0.187, *p* < 0.01), attachment (*β* = 0.295, *p* < 0.01), and identity (*β* = 0.280, *p* < 0.01) have a significant positive impact on community commitment. Thus, hypothesis 6, 7, and 8 are established.

**Figure 3:**
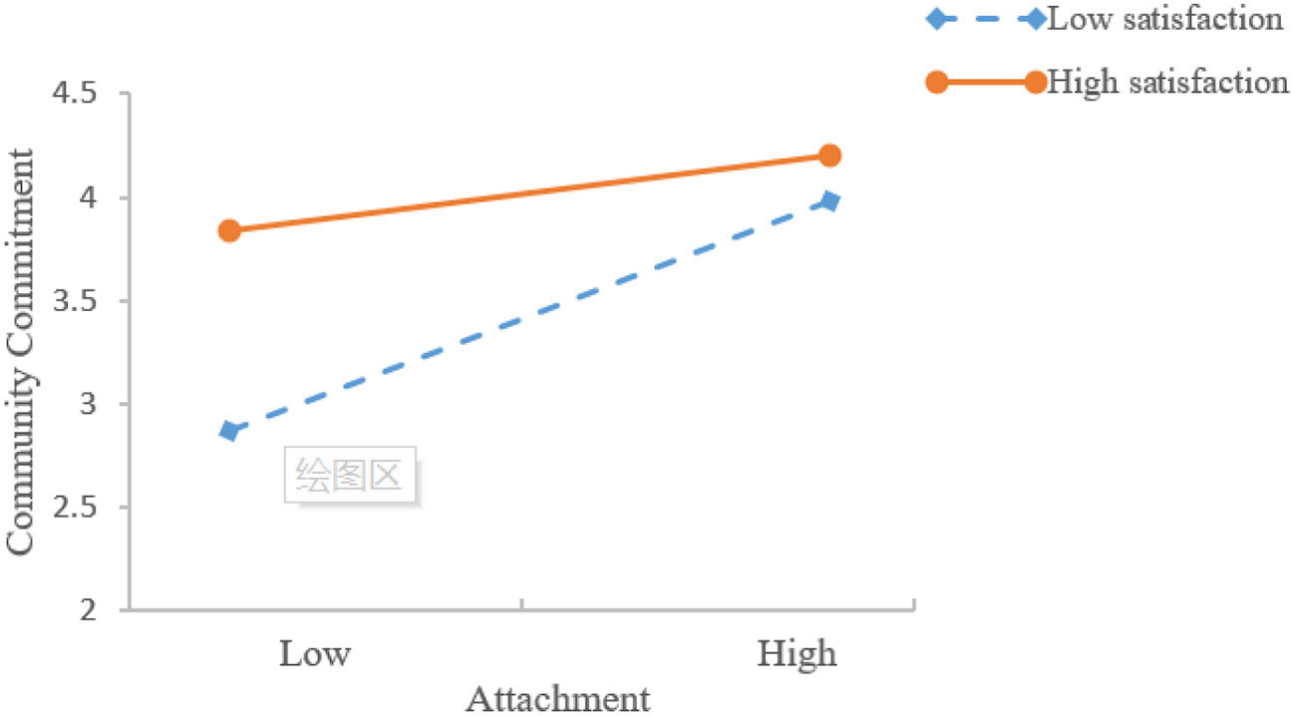
Interactive effects of satisfaction and attachment on community commitment.

**Figure 4:**
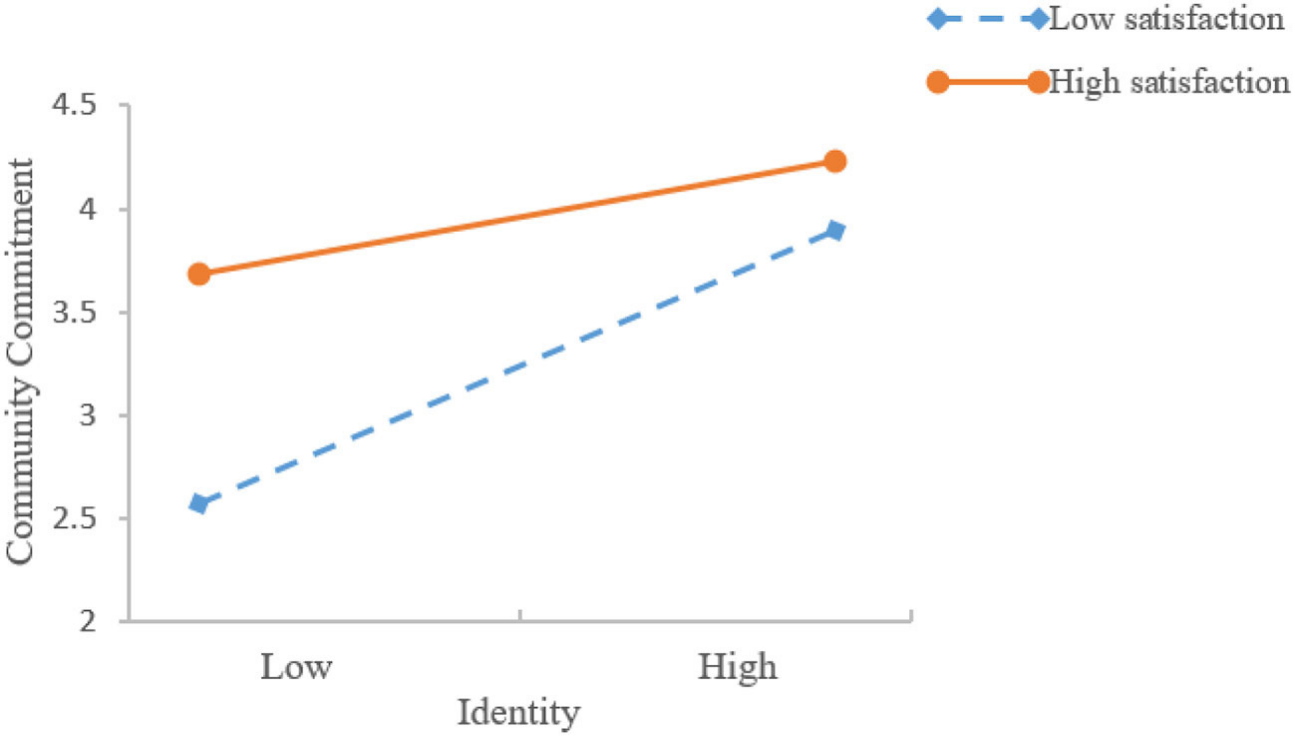
Interactive effects of satisfaction and identity on community commitment.

In order to test the interaction supplementary relationship, this paper constructs the interaction items of satisfaction and attachment, satisfaction and identity, respectively, and adds them to the model. It can be seen from Table 3 that the interaction between satisfaction and attachment is significant (*β* = −0.141, *p* < 0.05), indicating that under low satisfaction, attachment will have a supplementary and reinforcing effect on the community commitment of user, hypothesis 7a is supported. At the same time, Table 3 also shows that the interaction between satisfaction and identity is significant (*β* = −0.168, *p* < 0.05), indicating that under low satisfaction, identity will also have a supplementary and reinforcing effect on the community commitment of user. Hypothesis 8a is supported. In order to more intuitively demonstrate the interactive complementation, this study conducted a simple slope test. That is: use the mean value of satisfaction plus or minus one standard deviation as a grouping criterion to describe the relationship between attachment, identity, and community commitment at high and low levels of satisfaction, respectively. The specific results are shown in Figures 3, 4. It can be seen from Figures 3, 4, although when the satisfaction is low (*β* = 0.7251, *p* < 0.001) and high (*β* = 0.2385, *p* < 0.001), attachment feeling has a significant impact on community commitment, but through comparing the impact coefficients, it can be seen that the impact of attachment on community commitment is significantly greater when satisfaction is at a low level than when it is high. This further shows that when the satisfaction level is low in the virtual knowledge community, attachment feeling can supplement and strengthen the impact of satisfaction on community commitment, so it becomes a useful supplement to satisfaction. Similarly, when satisfaction is low, identity has a strong positive effect on community commitment 10 (*β* = 0.6855, *p* < 0.001). When satisfaction is high, identity has a weak positive effect on community commitment influence (*β* = 0.2064, *p* < 0.001). This further shows that when satisfaction is low in the virtual knowledge community, identity can make up for the lack of satisfaction to the community commitment, and thus become a useful supplement to satisfaction.

## Discussion

The findings of this study reveal that both knowledge-seeking motivation and entertainment- seeking motivation can positively influence social-interaction motivation. This result means that knowledge-seeking motivation and entertainment-seeking motivation at the situational level enhance the social-communication motivation at the contextual level. It can be seen that knowledge-seeking motivation and entertainment-seeking motivation have no direct impact on community commitment. Knowledge-seeking motivation and entertainment-seeking motivation are the only primary motivations for users to participate in the virtual knowledge community. Knowledge-seeking motivation and entertainment-seeking motivation only act on higher levels of motivation will ultimately affect the community commitment of users. This also provides an explanation from the conclusion of Jang et al. (2008) that the motivation level is that “information quality has no significant effect on community commitment.” At the same time, by comparing the path coefficients of knowledge-seeking motivation and entertainment-seeking motivation, it can be found that knowledge-seeking motivation has a greater positive effect on social-communication motivation than entertainment-seeking motivation. In other words, in the virtual knowledge community, although the social-communication motivation of users is subject to the common role of knowledge-seeking motivation and entertainment-seeking motivation, knowledge-seeking motivation plays a leading role in the process of promoting social-communication motivation. This is consistent with the service purpose and communication content of the virtual knowledge community.

The analysis results show that there is a positive correlation between social- interaction motivation and self-efficacy. This result provides empirical support for the “relevant effect of contextual-level motivation on personality-level motivation” proposed by the motivation level model, that is, the social-interaction opportunities at the contextual level improve the personality level of self-efficacy (Vallerand, 1997). It also validates the opinion of Bagozzi and Dholakia (2006a) to a certain extent, that “the formation of community commitment is the result of the continuous internalization of users' participation motivation under the influence of external situation factors and personal psychological needs.” At the same time, we found that self-efficacy also has a significant positive effect on satisfaction. This conclusion is consistent with the research results of Hsu and Chiu (2004) that self-efficacy has a positive effect on satisfaction with the use of electronic services.

The findings show that satisfaction has a positive effect on attachment and identity. This result is intrinsically consistent with the research conclusion of Hashim and Tan (2015), that is, in the virtual community, the emotions (attachment, identity) of the user play an intermediary role between satisfaction and the willingness of the user to continue to contribute knowledge. That is to say, users who are satisfied with the virtual knowledge community will have a sense of attachment and identity to the community, and are willing to maintain long-term contact with the community. At the same time, the results of this study show that satisfaction, attachment, and identity can all positively affect community commitment. This result confirms that integrated emotion has a strong influence on user behavior intention. As Park et al. (2006) pointed out, under the influence of strong emotions, users will be willing to spend more time and energy to stay in touch with the community. It can be seen that multiple integrated emotions play a crucial role in community commitment. This conclusion further validates the attachment decision theory, that is, the individual attachment intensity and user commitment behavior intention depend on autonomy needs, relationship needs, and ability needs. These three needs coincide with the motivation needs of the three levels in this study. It can be seen that satisfying these three needs can enhance the satisfaction, attachment, and identity of the user, and then promote the behavioral intention of the user to maintain a long-term relationship with the community. The above results provide a stronger explanation for the promotion of community commitment of users by integrative emotion. In addition, the results show that attachment and identity can complement and strengthen the community commitment of users under the condition of low satisfaction. That is to say, in the virtual knowledge community, when the satisfaction is low, attachment identity can make up for the insufficient effect of satisfaction on community commitment, and thus become a useful supplement to satisfaction. This result has obvious internal logical consistency with the research conclusion of Haumann et al. (2014), that is, whether at the level of time or the level of the competitive environment, compared with satisfaction, the impact of attachment and identity on user behavior intention is more stable.

## Conclusion

In the context of the virtual knowledge community, based on the analysis of multiple intermediaries and moderating effects, this paper builds a relationship model of cross-level motivation, integrated emotion, and community commitment, and explores the role mechanism of cross-level motivation and multiple integrated emotions on virtual knowledge community commitment and verified the theoretical hypothesis of this article through empirical research. The research shows that: (1) The motivation to promote virtual knowledge community commitment has a hierarchical character, and follows a hierarchical structure that influences each level from the situational level to the personality level. Knowledge-seeking motivation and entertainment seeking at situational-level opportunities enhance the social -interaction motivation of users at the contextual level, and further improve their self-efficacy at the personality level, which ultimately helps promote the commitment of users to the virtual knowledge community. (2) The user has a wealth of integrated emotions for the virtual knowledge community, namely satisfaction, attachment, and identification. This multiple integrated emotion model is conducive to promoting the community commitment of the user. (3) In the virtual knowledge community, attachment, identity, and satisfaction have significant interactive supplementary effects. Attachment and identification are beneficial supplements to the impact of satisfaction on community commitment. That is, when satisfaction is low, attachment and identification can complement and strengthen the commitment of community.

### Theoretical Contribution

The theoretical contribution and innovation of this article are mainly reflected in three aspects.

First of all, this article focuses on a special kind of community such as a virtual knowledge community. It conducts research on the community commitment of its users, implements detailed research on virtual communities, and deepens the theoretical analysis of community commitment. At the same time, this article focuses on the joint impact of motivation and emotion on community commitment. Using “motivation-emotion-community commitment” as the main framework, a relationship model of cross-level motivation, multiple integrated emotions, and virtual knowledge community commitment is built, thus breaking the research limitations that separate the influence of motivation and emotion provide a theoretical framework for a deep understanding of the relationship among motivation, emotion, and community commitment.

Second, based on the motivation level model, this paper finds that the motivation of users in the virtual knowledge community has hierarchical characteristics, not just a single concept or different types in the same dimension. It also pointed out that the influence of various levels of motivation on community commitment follows the law of progression from the situational level to the personality level, which expands the application space of the motivation level theory to a certain extent, and opens up new ideas for the future research on motivation of virtual knowledge community commitment.

Finally, different from previous studies that only used satisfaction as an influencing factor of community commitment, this paper introduces multiple integrated emotions to carry out related research, points out that attachment, identity, satisfaction, and other integrated emotions all have a positive impact on virtual knowledge community commitment, and finds that there is an interactive complementary role between integrated emotions. On the one hand, this study deeply explains the complex mechanism of action between integrated emotions, which enriches the content system of integrated emotion theory; on the other hand, it broadens the analytical horizons of the emotional factors of community commitment, and thus provides a new entry point for the research on the mechanism of the effect of community commitment.

### Practical Implications

Based on the above research conclusions, the author believes that improving the commitment of the virtual knowledge community should start from the following aspects.

First, the community should strive to establish a complete knowledge information base and publish interesting exchange topics to attract users to join and stay for a long time. From the subject of this research, we can see that the initial motivation of users to enter the virtual knowledge community is based on knowledge seeking. Therefore, in order to meet the knowledge needs of users, the community should establish a relatively complete knowledge information database to maintain the knowledge richness and accuracy, provide users with reliable and useful information in time, thereby improving information search efficiency of the user, and providing knowledge support for users to learn. At the same time, in order to meet the entertainment needs of users, we can consider to establish an interesting knowledge exchange section, or encourage community users to publish interesting knowledge posts by combining physical rewards, virtual points rewards, and spiritual rewards, so that users can acquire relaxed and pleasant psychological experience while learning knowledge, so that they are more willing to maintain a long-term relationship with the virtual knowledge community.

Second, the community should strengthen external rewards and internal incentives to promote user social interaction and enhance self-efficacy. On the one hand, community managers should adopt multichannel strategies to encourage user interaction, including carrying out rich online interactions and rewarding active users. Specifically: First, the community can encourage users to improve their own information through corresponding virtual point rewards, so that users can understand and become familiar with each other, thereby promoting users to build their own friendship circle; Second, a network screening system can be set up to recommend the corresponding information and members according to the content of browsing of users, the interaction between users and the similarity of personal interests, so as to develop their personal relationship network; Finally, establish a community circle based on the interests and hobbies of users, provide an interactive platform for like-minded users, and create a good social atmosphere. On the other hand, community managers should satisfy the pursuit of prestige and their own value of users through various ways, such as: providing user level identification, reputation tracking mechanism, and value display of user posting content; Through measures such as promotion of ranks and granting more operation authority, to meet the pursuit of prestige and their own value of community members; Users of different levels can also be given certain participation and management privileges to encourage them in terms of community recognition and empowerment. They can also recommend influential “opinion leaders” to manage community activities and increase the sense of self-efficacy of participants.

Third, the community should aim to establish a strong emotional connection mechanism and pay attention to fostering a deeper integrated emotion for users. First of all, the virtual knowledge community platform should strengthen the release and supervision of information, and do a good job of providing users with a large number of high-quality knowledge and information; set up a dedicated interpersonal relationship manager to regularly remind users to connect and communicate with each other; carry out various thematic discussion activities from time to time, and distribute red envelopes to super contributors and users with long participation time. Through the above measures to meet the diversified motivations and needs of users, and increase user satisfaction with the virtual knowledge community. Second, by continuously strengthening the service functions of the community, creating a good community environment and communication atmosphere, we strive to make the virtual knowledge community an indispensable part of the daily lives of users, and form and enhance the attachment of users to the community. Third, various offline activities can be held regularly to open up different channels of knowledge exchange and play the synergistic effect of various channels to enhance the sense of community identification of users through more efficient communication. In short, we should use various methods to enhance the emotional support function of the community, give full play to the interactive supplementary role of integrated emotions, so that the virtual knowledge community maintains a healthy and sustainable development.

### Research Limitations and Prospects

Due to the limitations of data and models, this article still has the following areas to be improved and expanded. First, although the sample size of this study meets the requirements of statistical analysis, the data size is still relatively small. In the future, the author will expand the sample size to further confirm the experimental results of this study. At the same time, although the method of collecting data in this study collects most virtual knowledge communities, it does not cover all knowledge communities, which limits the scope of sampling to a certain extent. We recommend that scholars with sufficient resources include the wider community to improve the integrity of future research results. Second, due to space limitations, this study did not study the individual differences of users. In fact, gender differences, personality characteristics of users, self-expression tendency, and other individual factors will also have an impact on the community commitment of users. Therefore, it is necessary to investigate the individual characteristics differences in the follow-up research. Third, virtual knowledge community is only one kind of online community, and there are many other types of online communities, such as online medical community, online entertainment community, online brand community, etc. Follow-up research will consider more types of virtual communities, and further explore the mechanism of user community commitment and the differences of different communities, so as to improve the integrity of future research. Finally, the virtual knowledge community has a dynamic development process. The follow-up research can further explore how the participation motivation, integration emotion, and community commitment of user interact in different development stages of the virtual knowledge community from a dynamic perspective. In short, in future research, the author will strive to analyze the influencing factors and mechanism of the virtual knowledge community commitment at multiple levels and from multiple angles to expand the research scope and related conclusions of this topic.

## Data Availability Statement

The datasets generated for this study are available on request to the corresponding author. Requests to access the datasets should be directed to Bing Sun, heusun@hotmail.com.

## Ethics Statement

Ethical review and approval was not required for the study on human participants in accordance with the local legislation and institutional requirements. The patients/participants provided their written informed consent to participate in this study.

## Author Contributions

BS, HM, and MK contributed conception and design of the study and wrote sections of the manuscript. MK organized the database. HM performed the statistical analysis and wrote the first draft of the manuscript. All authors contributed to manuscript revision, read, and approved the submitted version.

## Conflict of Interest

The authors declare that the research was conducted in the absence of any commercial or financial relationships that could be construed as a potential conflict of interest.

## Publisher's Note

All claims expressed in this article are solely those of the authors and do not necessarily represent those of their affiliated organizations, or those of the publisher, the editors and the reviewers. Any product that may be evaluated in this article, or claim that may be made by its manufacturer, is not guaranteed or endorsed by the publisher.
